# Older adults’ perceptions of online physical exercise management

**DOI:** 10.3389/fpubh.2024.1303113

**Published:** 2024-02-06

**Authors:** Judita Peterlin, Vlado Dimovski, Simon Colnar, Bojan Blažica, Anamarija Kejžar

**Affiliations:** ^1^Unit for Management and Organisation, School of Economics and Business & MRRC UL SI-AHA University of Ljubljana, Ljubljana, Slovenia; ^2^Computer Systems Department, “Jožef Stefan” Institute & MRRC UL SI-AHA University of Ljubljana, Ljubljana, Slovenia; ^3^Faculty of Social Work UL & Unit for Management and Organisation, School of Economics and Business & MRRC UL SI-AHA University of Ljubljana, Ljubljana, Slovenia

**Keywords:** ageing, older adults, online physical exercise, focus group, health management, age management

## Abstract

The study addresses the challenges of digitally transforming physical exercises for older adults (aged more than 55 years) to be performed in virtual environments (during the COVID-19 pandemic) as a long-term proactive strategic initiative in response to the global ageing society and technological development trend. A focus group with a sample of 24 older adults and three trainers were used as part of a 3-month physical exercise pilot conducted by the Jožef Stefan Institute to identify the skills and well-being gained and identify factors that influence success with online exercises for older adults on the individual and organisational levels. First, on the individual level, communication differences were identified when comparing face-to-face exercises with online exercises. Second, on the organisational level, the study identified several challenges arising from the digital transformation of exercises (i.e., onboarding, technical, structural, isolation and motivational). Finally, recommendations are proposed to transform older adults’ exercises when performed in a virtual environment. The study results can also benefit health management practices and theory in the work environment to ensure that older workers can still utilise their strengths to perform successfully while remaining healthy. Online physical exercises tailored to older adults’ needs and specifications could be provided as part of corporate wellness programmes in organisations.

## 1 Introduction

Approximately 31% of the global population aged ≥15 years does not engage in sufficient physical activity, with this being known to contribute to the death of approximately 3.2 million people every year (1). A sedentary lifestyle is becoming one of the biggest health problems of modern times. However, the large number of deaths is not the only problem. A bigger problem is the significantly lower quality of life caused by excessive sitting. Frequent inflammations and non-communicable chronic diseases prevent the individual from performing at a high level in almost all areas. At the same time, they are also the main burden on health insurance. On the other hand demographic projections suggest that by 2060 every third inhabitant of the Republic of Slovenia will be 65 years of age, and almost every seventh person will be older than 80 years (2). Trends show that the working population of older workers in EU-27 will grow up to 16.2% (to 9.9 million) from 2010 to 2030 (3). This means that employers must use digital tools that enable physical exercise to make older employees fit for work. The World Health Organization [Strojnik as cited in (4)] recommends 30 min a day of moderately intense activity.

Sport for senior citizens can be defined as a sport and recreational activity of people aged over 65. This is usually a period when people retire, due to ageing and changes in certain biological characteristics of the human body, so older adult people fall ill more often. For society, sports for senior citizens may represent an important means to reduce expenditure on the public health fund, as the effects of quality physical exercise reduce morbidity. And, for an individual, regular sports activity during this life period means sound and independent ageing, maintenance of physical, mental, emotional, and social health, social inclusion, reduced treatment costs and creative life energy (5).

One major challenge during the COVID-19 pandemic was the limited accessibility to healthcare facilities, especially for the older population. During the COVID-19 pandemic, both healthcare stakeholders and the older population claimed that the healthcare needs of the older people and their caregivers increased dramatically in all countries, especially in Italy (Calabria), Croatia and BiH. The results of the study took place in the following countries: Slovenia, Italy (Calabria), Croatia, Bosnia and Herzegovina, Greece, Montenegro, and Serbia. Overall, 722 older people and 267 healthcare stakeholders participated in the study. According to the results, countries from the Adrion/Ionian regions faced significant challenges to adjust to the special needs of the older people during the COVID-19 pandemic, which was possibly due to limited accessibility opportunities to healthcare facilities. These results highlight the need for the development of alternative ways of providing medical assistance and supervision when in-person care is not possible (6). Immediately following the outbreak of the COVID-19 epidemic, the older adults in Slovenia were also encouraged to be physically active in new ways. Televised home physical activity – strength, flexibility, and range-of-motion exercises led by qualified physical education teachers – were broadcast on national television from 18:00 daily. This activity was just one of several components of the national campaigns #vadidoma, and #trenirajdoma, initiated by SLOfit and organised by the national TV broadcaster, the Slovenian Olympic Committee (SOC) and the Faculty of Sport at the University of Ljubljana. The campaign was intended to reach the largest and most diverse audience possible and online platforms/ physical activity lessons were livestreamed on Facebook. The SOC also prepared a series of promotional clips with top athletes encouraging people to continue exercising at home during the period of isolation (7). This promotion of various sports activities online due to the epidemic also gave employers the possibility for the long-term implementation of innovative programmes to promote employees’ physical activity.

In several countries, legislation requires employers to take care of health in the workplace. It is estimated that in Slovenia about one-third of companies comply with this [Strojnik as cited in (4)]. Individual campaigns to promote health in the workplace are led by various bodies (chambers, institutes, ministries). For example, the Slovenian Act on Safety and Health at Work states that the employer must plan and implement health promotion at the workplace, providing the necessary resources and methods for monitoring its implementation (8). This means that it carries out certain activities and measures with the aim of maintaining and strengthening the physical and mental health of workers. Employers’ awareness in this area is also growing. Individual companies take care of promoting health in the workplace in a very planned manner. They organise various exercises and physical activities for employees or connect with external providers such as sports clubs and fitness centres. They organise a healthy diet (e.g., free fruit on certain work days at the office; vitality meal options at the canteen; having a kitchen available in the workplace etc.), courses on correct ergonomic handling etc. Along these lines, there is also ever more information and communication support, making it easier for companies to organise such activities. For employers, the health of employees is associated with a direct financial benefit for the company [Strojnik, as cited in (4)]. The positive effects linked with the implementation of health and well-being and the promotion of employee well-being in the work environment are numerous. According to EU-OSHA (9), the promotion of health and well-being in the work environment contributes to the better health and well-being of employees. Data from Eurofound (10) show that the employees’ health and well-being have a significant impact on the performance of organisations by reducing medical absenteeism and turnover, increasing employee satisfaction etc. The Eurofound (10) research results also show that in many organisations which made such efforts employees had better health and well-being and their productivity rose by up to 20 percent (11). A good practice of a Slovenian insurance company confirms the value and positive effects of health promotion through the project Promoting workplace safety and physical and mental health in an insurance company. Zavarovalnica Triglav, d.d. is a multinational insurance company. Its headquarters are located in Ljubljana, Slovenia, with subsidiary offices in Bosnia and Herzegovina, Croatia, Montenegro, North Macedonia and Serbia. In June 2021, the company had 2,250 employees. The company wishes to create conditions for its employees to be healthy and satisfied at work, to feel part of the company, to strive for personal development and take care of their health as an everyday habit, both in the office and while working from home. The many activities in the project include Active breaks – an exercise session run by physical education teachers twice a week online. These activities achieved: a decrease in sick leave between 2019 (23%) and 2020 (19%). Three hundred employees participated in Active breaks (9).

Older adults are faced with modern technology, where some know how to use it and others have great difficulty even using a smartphone. To remain integrated into society, mobility plays an important role. Older adults are confronted with changing mobility vehicles, and ‘micromobility’ around them that demand them to be fit and vigilant concerning how they move, given that it is very important that they can move and be safe in the world around them. Evidence shows that older adults and disabled people feel less safe in today’s traffic than prior to the introduction of micro-mobility (12). If older adults train and maintain their physical fitness regularly, they are better equipped to use modern technology and ways of transport that are also sustainable for managing daily tasks, such as shopping for groceries or visiting a friend, and remaining socially and physically active.

The use of new digital technologies to support an improvement in processes and services is called digital transformation (13). A trainer and the older adult workout participant compose a team with a shared goal the older adult must follow. The instructions of the leader (trainer) are especially important if the older adults has any injuries or a particular health condition. With the goal of bringing exercises closer to older adults who are located geographically apart (e.g., in a rural setting), online physical exercises provide an alternative to keeping up their physical condition. Online exercises are exercise arrangements in which geographically dispersed participants with limited face-to-face contact exercise independently using electronic communication media to achieve a common goal. This definition is in line with Dulebohn and Hoch’s (14) definition of virtual teams. Digital transformation opens new possibilities for processes, products, innovations, and the growth of services (15). However, transferring the tacit knowledge which can spontaneously happen in face-to-face interaction over medium-lean online communication channels proves to be more difficult (16). Studies that addressed knowledge creation and transfer (17) and loss (18) researched conventional organisations and teams. Further, studies of virtual teams tended to focus on communication problems arising from geographical and cultural differences (19). Communication, knowledge transfer, and learning in the context of the digital transformation of a team were addressed in a research study by Vuchkovski and associates (13).

Our research questions stem from the work of Vuchkovski et al. (13) and are as follows: (1) How have communication, knowledge transfer, and learning changed in the trainer–participant interaction due to the digital transformation of physical exercises for older adults? (2) Which challenges arise from the lack of technological knowledge and skills (on the sides of both the trainer and/or older adult participant) in connection with older adults’ physical exercises? (3) Which individual and organisational skills are required to successfully manage a physical workout in a virtual environment? (4) Which (new) roles of trainers and older adult workout participants emerged as a result of the online physical exercise instruction?

Our research is not only timely but also relevant to most organisations today. The results of this study may be of particular interest to companies that have yet to incorporate age management and health management in their corporate culture and considering to do so in the future. In addition, the study will help human resource managers understand the challenges of online workout programmes for older adults before adopting them in the future.

## 2 Theoretical overview

Kodama (20) states that organisations should focus not simply on the implementation of digital technologies but also on the skills required to use new technologies. Accordingly, trainers in the future wishing to train their older adult participants online will need to invest time also in teaching their participants digital skills besides developing the physical exercises. Digital transformation strategies (21) have four dimensions in common: (1) use of technologies (the attitude to new technologies as well as the organisation’s ability to use these technologies); (2) changes in value creation (society’s and the organisation’s attitude to new technologies as well as the organisational ability to use these technologies); (3) structural changes (changes in the organisational setup); and (4) financial aspects (ability to finance a digital transformation). Conducting physical exercises online calls for certain adjustments and in this paper we are interested in determining which challenges exist on the side of trainers and which challenges participants, especially older ones, need to overcome to accomplish their workout goals. The physical exercises of older adults involve several stakeholders (Table 1).

**Table 1:** Perspectives of stakeholders.

Stakeholder	Key interest in physical exercises for older adults
Older adults	Wellness, physical fitness, social health
Trainers	Appropriate training, quality of the implementation
Clinicians	Safe implementation, health management
Managers	Raising fitness goals, recruiting and training qualified older workers, segmenting different groups of exercises, a decrease in absenteeism, organisational culture
Economists	Capital and operating costs of injuries of the older adults, human capital requirements and development of older adults
Behavioural scientists	Older adult workers’ risk behaviours, ergonomics, safety and training
Policy scientists	Safety policies, age management, policies, health management policies
Politicians	Fit, healthy, skilful, productive older adults who are integrated into society (legislation, regulation)

Source: Adapted after (22), p. 9.

The nature and capacities of implemented processes reflect organisational investment decisions. Namely, the greater the investment (pretesting of participants, e.g., psychological testing, orthopaedic testing, ICT skills testing, cognitive testing…), the more detailed the processes, the better fit of the execution of exercises that are tailor-made to suit participants’ needs. Society’s values and norms regarding investments in training, maintenance and safety are key to how exercise for the older adults is implemented [(22), p. 7]. Our goal is to ensure that all the ingredients for model-based failure management are clear and that the recipe for successful exercise is compelling [(22), p. 8]. Change depends on both planning (vision, strategy) and leadership [communication; (22), p. 8].

### 2.1 Online physical exercise

Regular physical activity brings several health benefits that are well documented in the literature (23). Despite this, around the world the lack of physical activity is a major risk factor for diseases. While this description applies to the general population, it is even more detrimental for older adults and individuals who are already dealing with chronic disease (24). In 2020, during the period of the coronavirus outbreak, the World Health Organization (1) recommended that all adults attend some sort of online physical activity so as to improve their total daily activity levels. Similarly, Ricci et al. (25) recommended that adults join online physical activity exercises given that are particularly suitable for positively influencing an individual’s endurance, strength, flexibility and balance.

Building on the study by Schwartz et al. (26), we support the claim that delivering live or recorded, virtual, group training exercises via video-conferencing solutions is a viable way of boosting older adults’ levels of physical activity. Moreover, it may be assumed that online exercises can help older adults with initiating and adhering to a physical exercise routine. Online physical exercise today benefits older adults by virtue of the greater availability and popularity of technological devices and smart phones among them, since they are a necessary precondition for video-conferencing interfaces that enable online physical exercises (27). One may argue that today many older adults are online and wish to be present there (28). Contemporary research suggests that in advanced economies such as the economy that was considered in this research a high percentage of older adults actively uses the Internet (29). We thus claim that technology-based exercises can enhance the physical activity of older people, which allows them to feel empowered and have greater self-esteem. Smart technology solutions are well-accepted methods that permit older adults to take exercise programmes, which positively influence their health, psychological outcomes, a significant number of clinical parameters, motivation, and levels of enjoyment. New and modern technological solutions such as Fitbit are gaining recognition as a viable alternative to conventional face-to-face exercising (30).

Nevertheless, despite the considerable promise held by online exercises, we must also explore their overall feasibility among older adults since they are accompanied by unique challenges related to specific characteristics of older adults. Looking from a safety perspective, there is the potential for the occurrence of a fall that can bring serious consequences for the overall well-being of an older adult. We must also take the potential physical impairments of older adults into account, such as the hearing or vision difficulties often found among this part of the population (26). Another challenge is how to engage the most vulnerable older adults who do not have access to online sources, such as individuals with a low income and lower education. Digital skills and knowledge among the older adults population can vary significantly (28). Some older adults possess exceptional digital skills while others only have basic digital skills (31); in theory, this is labelled the “grey digital divide.” According to Friemel (32), this digital divide is explained by technical issues, physical constraints, potential cognitive impairments and psychological issues. When examining physical exercise in an online environment, it is also worth noting that women tend to be more open to participating in such activities that include exercise, health and well-being (33).

### 2.2 Health management

Unhealthy behaviours such as a lack of physical activity represent risk factors for negative health outcomes (34) or, in the case of older employees, can be reflected in lost productivity or absences due to sickness (35), even as a reason for an early exit from the labour market (36). In contrast, empirical research shows that health promotion programmes lowered absenteeism in the workplace and even employer costs, while simultaneously increasing the productivity and health of employees (37). Halling Ullberg et al. (38) report a decline in cardiorespiratory fitness among adults. As stated by Demou et al. (39), workplace health promotion could follow the route of greater flexibility in the time and location of specific programmes and activities, which might also offer support with online physical exercises by offering flexibility in arranging them. Since we are dealing with the challenge of how to prolong the working career of older employees, there is also the need to remain healthy to be able to continue to be an active part of the labour market (40).

The working environment is in fact a promising context for health promotion given that older employees spend a large proportion of their time at work and with the help of existing social networks this can trigger a change in behaviour towards a healthier lifestyle. Consequently, a number of health promotion programmes were already offered in the past in the working environment (40). Drake et al. (41) argue that a physically active life is associated with better health behaviours.

### 2.3 Knowledge transfer

In today’s fast-paced and ever-changing work environment, older adults must strive towards lifelong learning and seek new knowledge, which is gaining in importance for their progress and success (42). Knowledge transfer is a process of knowledge exchange between stakeholders in which newly gained information can be utilised in a range of ways (43). Knowledge transfer includes the acquisition, communication, implementation, acceptance and assimilation of knowledge and know-how (44). While exploring the knowledge transfer process, we must consider the overall quantity and quality of information that is part of the transfer because it defines the content of the knowledge (45). Knowledge quality has a close correlation with the perceived usefulness for individuals (46). Knowledge transfer as part of knowledge management has historically attracted considerable interest from the scientific community and practitioners (47). Particular interest is paid to the question of which antecedents and characteristics enhance the quality of knowledge transfer (48). As such, knowledge transfer enables individuals to develop the ability to do activities differently (49) by way of a cognitive process that provides them with awareness of their learning capabilities. Further, knowledge transfer is a process where knowledge is transferred both directly and indirectly (45). In this process, a relationship between the source of knowledge and the recipient of knowledge develops (50).

When it comes to online physical exercise, we can also argue that it can benefit from one of the well-known benefits of virtual environments that enables individuals access to knowledge via information communication technologies. Knowledge transfer in this context can occur from individuals (trainers) to individuals attending an online exercise session regardless of their physical location (51). Also important is the constant collaboration between different stakeholders that ensures that all interests are considered and appreciated, which then corresponds with a better knowledge transfer (52). Even though we are in an online environment, social interaction plays an important role as it can speed up and positively influence specific knowledge transfer (53). However, with the latter very specific example we must mention that it is important to develop a unique model of transferring knowledge, where a positive environment that supports continuous learning and knowledge transfer is an important enabler (54). Duryan et al. (54) posit that knowledge transfer is possible via video conferencing and other online solutions, albeit they believe that this can be considered as passive.

The transfer of knowledge positively influences individuals’ capabilities to engage in specific activities such as in our example online physical exercises, as well as their skills and expertise on a topic of interest (55). Similarly, Tangaraja et al. (56) claim that knowledge transfer can occur using online methods that include technology intervention. In addition, as actual knowledge transfer occurs between individuals that may have mixed levels of motivation, willingness and ability to engage in knowledge transfer (57), we must acknowledge that in the example of older adults participating in online physical exercises those levels might significantly vary from one individual to another. Gaur et al. (58) similarly state that successful knowledge transfer relies on several factors pertaining to individuals. Empirical studies show that knowledge transfer has a positive impact on knowledge quality, which influences user satisfaction (59), which also implies that knowledge transfer might have an important role in user satisfaction with online physical exercise.

### 2.4 Age management

Active ageing is typically defined as a policy initiative to counteract the negative effects of population ageing. The key is to find appropriate solutions to extend the working lives of individuals by encouraging them to participate longer as active members of the labour market. Age management practices in organisations also have an important part in this aspect (60). Ilmarinen (61) defined age management as “managing the workability and organization of work from the viewpoint of people’s life course and resources whether the changes are caused by the aging process or other age-related factors.” Age management practices in organisations are growing in importance as they have a crucial effect on sustaining and to some extent even increasing the participation of older workers in the labour market (62). Although age management in general takes all age cohorts of employees into account, employees aged 50+ are the group most focused on in the field of age management in organisations (63).

**Figure 1:**
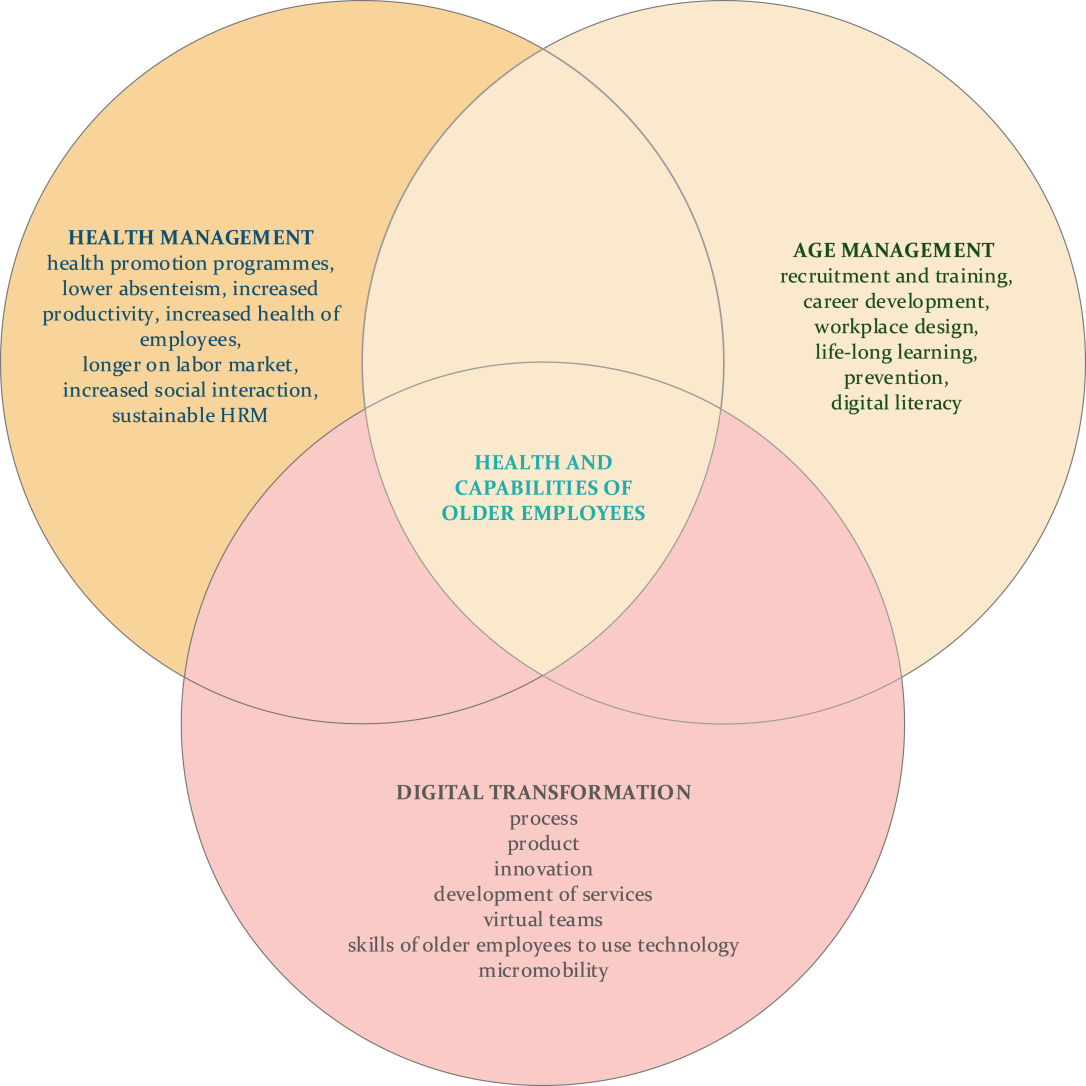
Age management in the workplace.

In the literature, there are five established dimensions for age management: (1) recruitment and training, (2) life-long learning and knowledge transfer, (3) career development, (4) flexible working practices, and health promotion and (5) workplace design (60). We argue that some health promotion activities can also include initiatives promoting online physical exercises for older employees. Organisations already have several activities available to promote healthier lifestyles, i.e., they could educate on diseases and present opportunities to prevent diseases, offer subsidies for sports activities (including online sports activities) and offer voluntary health examinations (60). Baruch et al. (64) argue that it is also in employers’ best interest to have older employees who are fit and healthy, albeit it remains somewhat unclear what the best pathway is for organisations to achieve this in practice (65). Similarly, on the individual level, health is an important factor as it is often an important characteristic in an individual’s decision-making process regarding whether to retire or continue working (66). Management must therefore find a solution to retain tacit knowledge and motivate older employees to stay committed to the firm (67) (Figure 1).

## 3 Methods

Qualitative data were obtained by observing the physical exercise sessions of 24 older adults (more than 55 years of age) over 3 months. We performed a focus group with 11 older adults who responded to our invitation and gave their written consent for participation out of 24 participants in the 3-month experiment. In addition, semi-structured interviews with two trainers were conducted in 2023. Focus group session was recorded with the agreement of the participants and lasted about 1 h. The focus group was transcribed and analysed by qualitative analysis in a process in which units (parts of sentences, sentences, whole paragraphs) of analysis were identified first, before open codes were defined for each part. Next, axial coding was performed (codes with similar meanings were grouped into categories and themes). Finally, relationships were established between categories and/or themes relevant to our research questions. Qualitative comparative analysis was used to assess the attitudes.

Validity in qualitative research (68) is traditionally determined as the degree to which the researcher’s claims regarding the acquired knowledge are consistent with the studied reality or the construction of the participants’ reality in the research process. A 28-year analysis of qualitative publications in management showed an evolutionary shift from an emphasis on the criterion of internal validity to external validity (69). External validity (70) establishes a domain in which qualitative research findings can be generalised. This domain is limited to the relevant conditions under which the findings were achieved. Construct validity is linked to properly established operational measurement mechanisms for the concepts under study. To achieve it, it is necessary to identify the studied phenomenon that corresponds to the theoretical concepts and then develop an appropriate measurement instrument that operationalises/determines the phenomenon.

Cho and Trent (68) build on two approaches – transactional validity and transformational validity – with a recursive, process-oriented alternative view of validity. Transactional validity approaches [(68), p. 321], such as interpretation, verification of participants in qualitative research, and triangulation, refer to the interactive process between the researcher, the researched and collected data, where a relatively high level of accuracy and consensus is sought through the revision of collected and interpreted facts, feelings, experiences, values and protections since it is assumed that transactional validity techniques will contribute to a more consistent, objective presentation of reality.

Triangulation refers to the investigation of a social phenomenon from different perspectives (71), whereby: (1) theoretical triangulation requires that more than one theory is used in the interpretation of the data; and (2) methodological triangulation expresses different dimensions of the phenomenon through the use of various methods. Triangulation is related to the accuracy of a single data unit, while participant verification (or participant validation) in research is aimed at the tentative credibility of the research (72).

We provided participant validation through participant feedback, which enabled correct descriptions and interpretation of the human experience such that people who have this experience immediately recognise it as their own. Participant validation is often used in the emotionalism research approach (73) where the authentic experience of the participants is valued so that the researcher’s findings are confirmed by the research participants. Reason and Rowan [in (73), p. 292] criticise researchers who fear “contaminating” data with the subject’s experience, arguing that good researchers go back to subjects with partial results and refine them in light of the subject’s reactions. Bloor (74) points out possible shortcomings of the validation of participants who have no interest in ensuring the validity of the research, do not understand the scientific/professional vocabulary, or the results of the analysis are incompatible with their self-concept. Fielding and Fielding [in (73), p. 293], in contrast, believe that research participants provide additional knowledge in providing validity in terms of the context of their performance, but should not hold a privileged status in the analysis of their performance. Yet, their feedback should not be perceived as a direct guarantee of validity or the rejection/abandonment of the researcher’s findings, but as an additional source of data and insight. Validity in the study of development processes is transactionally oriented in most cases and, if the purpose of the research is the resulting impact on organisational development, it is transformationally oriented [(68), p. 330].

The primary purpose of inter-rater reliability is to assess the extent to which different raters agree on their judgements or evaluations. This is critical in fields like research, education, psychology, and healthcare where subjective assessments are common.

To enhance inter-rater reliability, it is important to provide clear instructions, training, and calibration sessions for raters, which we carefully implemented during our research. These measures help ensure that raters have a common understanding and interpretation of the criteria/tasks.

In summary, inter-rater reliability was our crucial consideration because multiple raters were involved in making subjective judgements or assessments. It provided a quantitative measure of the oral agreement among the raters, thereby adding to the overall validity and trustworthiness of the data or assessments.

Regarding the small sample size, we should emphasise that the country in which the data were collected is small, we were trying to implement a novel approach, and succeeded in obtaining some of the first data that explain online exercise in our local context (Table 2).

**Table 2:** Levels of analysis.

Society	Society 5.0, ageing society, knowledge-based society, learning organisation paradigm
Organization	Jožef Stefan Institute (IJS), Fitness studio, Splošna bolnišnica Franca Derganca Šempeter, School of Economics and Business, University of Ljubljana
Processes	Online exercises Face-to-face exercises
People	3 trainers, orthopaedist, 24 participants (older adults), IT staff, IJS management team

Source: Adapted after (22), p. 7.

### 3.1 Goals for the (online) workout management of older adults

The goal of the pilot SI4CARE older adult physical exercises was to demonstrate the effectiveness and efficiency of the individualised exercise approach. Each participant was checked by an orthopaedist who set specific limits for participants if they had any existing injuries or operations behind them (75). The exercises were divided into three main parts. For the introduction, participants warmed up for 10 min (e.g., drove a stationary exercise bicycle etc.) and did stretching where the aim was to teach participants how to do the exercises correctly at home after the 3-month SI4CARE pilot finished. The main part encompassed exercises to activate the whole body, power and strength, as well as balance exercises. In the end, participants warmed down with walking and light stretching. If any participant came with the goal of losing weight, their exercises were further adjusted to that goal by providing additional exercises. The balance exercises advanced slowly, from less difficult to more demanding ones. In the first month, participants learned the basic exercises and the focus was on the correct technique of the exercise. In the second and third months, participants advanced and improved the techniques (76).

The pilot exercise experiment included 24 participants and 4 sports training and healthcare practitioners. The experiment was divided into three phases:

1. Preparation phase: Activities in this phase involved the preparation of documents for monitoring the pilot, preparation of the presentation for participants, and recruitment of the participants.
2. Implementation phase: The pilot was implemented in 4 batches of 6 participants, 2 online groups and 2 in-person groups, totalling 24 participants. Each batch consisted of: (1) an initial event with education, initial measurements and consultation with orthopaedists; (2) training programme development; (3) 2 x weekly training for 3 months in-person or online (once live with recording); and (4) final measurements and a satisfaction questionnaire. Each training session consisted of an introduction (10 min warm-up, 5 min dynamic stretching), the main training part (15 min trunk activation and balance exercises, exercises to increase sub-maximal leg strength); and a final part (10 min relaxing and warming down). During the 3-month intervention, the focus shifted from teaching proper exercise execution in the first month to increasing loads and difficulty in the second and third months.

Monitoring phase: After the pilot ended, we sent an online form asking all participants about their satisfaction with the activities. To quantify the acceptance of the activities, we used the net promoter score administered after the intervention. This is a widespread measure based on the simple question “how likely is a participant to recommend the service to a friend or colleague.” We included the participants as we had an experiment for 3 months and thus older participants meeting the rules of our sample were selected.

### 3.2 Participants and procedure

We carried out 1 face-to-face focus group with 11 participants of the SI4CARE pilot exercise. The average age of respondents who disclosed their age was 69.9 years (*N* = 11; one participant did not reveal their age; Table 3).

**Table 3:** Demographic characteristics of the focus group participants (*N* = 11).

Variable	N (%)
*Sex*	
Male	5
Female	6
*Education*	
Primary school	
High school	8
College/Faculty	3
*Living status*	
Alone	1
Partnered/community	9
Carer	1

### 3.3 Data analysis

Data analysis was first conducted by one of the researchers (JP) and subsequently by an independent researcher with experience in qualitative data analysis to add to the confirmability (AK). We performed inductive thematic analysis. The focus group transcripts were initially read several times. This process of immersion in the data is thought to serve as a ‘preparation stage’ before the actual analysis since it allows becoming familiar with the language and wording the participants used. Initially, first-order themes were identified within the response of each participant to the questions posed in the focus group. These themes were either directly related to the study’s research questions or entirely new topics that had emerged from the participants’ comments. In the next stage, these first-order themes were merged or clustered to form a second-order series of themes (“higher-order” themes or codes) based on the commonality of their meaning. In this stage, themes from the previous stage were either expanded to encompass others or ‘shrunk’ to become more specific. These final themes were more abstract in meaning than the previous ones and were the themes to be finally interwoven with the existing literature. The process described above was iterative as themes evolved and the data were better understood. Further reading led to the identification of additional themes not initially detected. Each researcher individually coded and categorised data from the same focus group to allow the triangulation of the findings. Data from the focus group was then coded by one researcher (JP) and were reviewed repeatedly by the other coauthors with particular attention paid to refining the codes proposed by all of the researchers. Through comparison, the researchers discussed and agreed on discrete themes. We refined and finalised the codes, resulting in a list of agreed themes (77).

## 4 Results

### 4.1 Management of introducing face-to-face physical exercises for older adults

People mostly sit, especially older adults in contemporary society. We sit at work, at home, and in our cars. Enabling older adults to be fit and have balance also allows them to move freely around with environmentally friendly vehicles, to have freedom (78), while saving time in traffic jams, saving money for fuel, and lowering the fear of navigating modern roads. Still, without physical fitness, one is hindered in terms of mobility. Our findings show the importance of physical exercise among older adults because it facilitates their mobility (without pain), social interaction, and confidence. Micromobility, such as an e-bike, is often used as a replacement for cars that some older adults are either afraid to use, or not allowed to use anymore, or have expressed that they cannot afford a new car. However, to use micro vehicles safely older adults need to pay attention to their remaining sense of balance, which is an integral part of physical exercises for older adults. Here, the online exercises proved to be more convenient for those participants who need knowledge about integrating into current traffic, whether they are afraid of new traffic trends, new vehicles, changed regulations, do not own a car, cannot hold a driving licence due to a special health condition (e.g., epilepsy) or are not allowed to drive anymore.

### 4.2 Barriers to older adults using (digital) technology

Technology is underdeveloped in rehabilitation, and older adults are not skilled enough to use modern technology fully (76). The supply of physical exercise (sports and rehabilitation) services targeted at the older adults is insufficient and does not meet the demand for such services, not even when taking both the public health system and private sector into consideration (79). According to the trainer Peter Krivec (76), who performed the exercise programme for older adults in the SI4CARE pilot, the advantages of exercising in the gym are: easier practice, more efficient learning of techniques, easier overcoming of mistakes, the possibility of using fitness appliances, no Internet connectivity issues, the possibility of technically more demanding exercises and quicker removal of mistakes, the possibility of socially gathering over coffee after the exercises are completed etc. A disadvantage for older adults taking exercises in the gym was having to travel to the location (76). Online exercises have the following advantages (76): the time component (no time is needed to drive to the location), convenient for the time of a pandemic (no physical contact allowed, lockdown), suitable for people with specific healthcare problems (epilepsy; no driving licence etc.). The disadvantages of online exercises are (76): lower level of quality in learning the appropriate techniques, lower level of rectifying mistakes in exercising, no fitness equipment available at home, and a smaller room for practice at home.

The older adults studied had similar exercise goals, with the majority expressing the need to keep in shape:

"*My goal was to get to know the exercises and then take advantage of them and ‘use’ them*" (F1; M1).

"*mastering the correct execution of the exercises*" (F1; M2).

"*improving flexibility and increasing my fitness”* (F1; M3).

"*An improvement in flexibility*” (F1; F1).

"*better mobility and balance”* (F1; F2).

"*flexibility and strength in individual joints*” (F1; F3).

"*greater mobility*" (F1; M4).

"*I want to maintain my fitness and make further progress*" (F1; F4).

"*body exercise and weight loss*" (F1; F5).

"*My goal was to become as elastic and flexible as possible*" (F1, F6).

According to one trainer (RR) engaged in the 3-month experiment, physical exercise for older generations is very important since it improves physical well-being, maintains muscle mass, and improves balance, which contributes to a better quality of life. Participants expressed appreciation and positive feedback regarding their improved abilities in everyday life. This meant it was easier for the older adults to carry out certain tasks during the day, such as mobility and everyday tasks. Due to the musculoskeletal problems some older adults had developed over the years, the progress made in improving their physical fitness varied. Some quickly mastered certain movements, others needed much more time. For visible progress, at least 3 months of training twice a week is required. The work programme for each individual was based on the performed biomechanical measurements, which showed asymmetries in the lower limbs. In addition, trainers took account of the previous problems and limitations of the participants (following a preliminary examination by an orthopaedist).

According to another trainer (TKP), people gain a lot physically and socially by exercising in their old age. With regular physical exercise, they avoid or at least delay the onset of various non-infectious chronic diseases and thereby maintain their functionality and independence, while also maintaining social contact with other older adults. The trainers objectively monitored the trainees’ progress through the initial and final torque measurements of the lower limbs, whereas during the training sessions participants also gave the trainers subjective feedback on changes in their well-being and abilities. For the first results to become visible, it takes about 4 to 6 weeks of regular exercise, but once again this depends on the individual.

### 4.3 (Dis)advantages and potential of (online) physical exercises for older adults

Overall, the pilot was perceived as great with a net promoter score of 60. The in-person condition was perceived even better, as excellent with a score of 78. With a score of 33, the online condition was less liked than the in-person one, yet still positively received. The older adults reported only minor technical problems with sound, while the trainers reported minor problems with Internet connection. Online exercise seems to be a good substitute for face-to-face exercise when there are special conditions, such as the lack of a possibility to come to the venue (no driving licence due to illness, age limit, no car, lockdown). Below, we present evidence by way of citations from the focus group members (F1, focus group held in the Nova Gorica region with 10 participants who practise online and face-to-face; F means a female and M a male participant):

"*The exercise took place online, an interesting experience, I learned a lot of new exercises. The emphasis was on the exercises ‘from head to toe’, holistically*" (F1; M1).

"*approach to and correct execution of exercises that I have not done correctly so far*" (F1; M2).

"*practise the method, expertise*" (F1; M3).

"*You exercised your body a lot during these exercises”* (F1; F1).

"*the personal approach and commitment to regular practice*" (F1; F2).

"*performing exercises under professional supervision, friendly trainers, weight loss*" (F1; F3).

"*the atmosphere, the professional delivery of the exercises*" (F1; M4).

"*the personal approach and training, I practised online. It was great, I practised from home. The only downside was that I had much older people in the group*” (F1; F4).

"*the exact display of exercise execution and warning about errors*" (F1; F5).

"*in general, I got to know a lot of exercises that I hadn't done before*" (F1; F6).

When we asked the focus group participants what they would change in their exercise if they were to start again with the exercises, they mentioned investing in technical information technology and equipment for better sound during the online exercises and being more active in doing the exercises. They also expressed regret over not having done those exercises years ago. They would welcome a holistic approach, by adding nutritional advice and regeneration approaches after the exercising, in a way a complete wellness lifestyle programme:

"*The problem was the sound, I would make sure of a quality speaker*" (F1; M1).

"*I'm sorry that I didn't take part in such organised exercises earlier*" (F1; M2).

"*more time during the exercises, something about nutrition during the exercises, regeneration after the exercises*" (F1; M4).

"*I would be more active*" (F1; F6).

Regarding the new leadership roles, our findings show that several new roles had emerged to effectively continue the processes in the virtual environment. In addition, multidimensional support (technical, organisational, even emotional) was introduced individually and within the workout teams (13). The experiences of the trainers are very positive as the older adults are a very grateful and warm-hearted group of trainees. Any progress or task they can do again on their own following the exercises is a very special victory and means a lot to them. Trainers adapted the training to the older adults by covering the main muscle groups, especially the torso and lower body. They included balance and reaction speed exercises because these functions decline with age. The programme itself was compiled in a simple and comprehensible manner, gradually, so that the trainees could learn and become aware of the movements.

### 4.4 Scepticism and mixed feelings about the online physical exercises for older adults

The preparation of the exercises itself went more or less without complications. However, during the exercises via the online platform, trainers and participants encountered the usual technical problems, such as a poor Internet connection and no picture or sound. This meant that sometimes they needed a little more time to explain and show the exercises. Otherwise, the online practice mode also proceeded fairly smoothly. After the live training, the trainees took their own initiative to regularly stop for coffee in the fitness bar which, unfortunately, the trainees did not have an opportunity to do via the online platform. Still, the online application was always open shortly before the start of the exercise session, and they could say a word to each other.

We asked employees in the current working environment about the challenges they encounter with organised physical exercises for the older adults in the virtual environment. They mentioned that the older adults may have less developed computer skills, making such exercise potentially more difficult to access. The correct execution of the exercises is questionable. Injuries a participant could acquire during the exercises (ankles, knees...) cannot be professionally assessed and treated by the trainer. An individual programme allows a person to follow the specific characteristics of the individual. An individual can choose the pace, load and exercises according to the needs and the desired goals the exercise should bring. Group exercise is more generic, providing a sufficient amount of movement and load, but it is not necessary for an individual to perform all of the exercises, especially among the older adults. Nonetheless, social and socialising aspects are extremely important in group exercises and lead to relaxation (79).

## 5 Discussion

Three shifts are called for in the health management field [(22), p. 175]: (1) organisations need to embrace interdisciplinary collaboration and move away from a “siloed work culture”; (2) decision-making needs to be data-driven instead of “experience-based leader-driven”; and (3) processes must become agile, adaptable and experimental instead of rigid and risk-averse. While designing exercises for the older adults to perform, productivity and safety must go hand in hand. This context determines who is recruited, how they are trained, and the nature of any incentives and rewards [(22), p. 179]. People often respond to subtle nudges (80). The older adults become accustomed to routine, ways of doing physical activity and thus technically skilled experts must possess the right motivational approach and communication to change the behaviour of older adults. Stakeholders (older workers, HR managers, employers etc.) need to see that the solution (physical exercise for older workers) being created will provide something of value to them [(22), p. 189]. Business processes must be well matched to a new market of older adults, where outsourcing is central to time to market due to the highly specialised skills required (physicians, psychologists, physiotherapists etc.), and the software processes needed to enable the migration of operating systems and apps across hardware platforms that older adults are used to and capable of developing new skills [(22), p. 84]. For health and age management to be implemented, the first step broadly sharing credible information so that all stakeholders understand the situation [(22), p. 188]. Data on older adults’ health, skills and training must be compiled and aggregated. It is necessary to involve professional stakeholders who value science, engineering and legal approaches to exploring solutions [(22), p. 188]. Public opinion holds that failures happen mainly due to external threats (81). Based on cases, sources, and studies of cases of failures, Rouse [(22), p. 194] argues that problems with failure management tend to arise from internal sources (imbalanced emphasis on operational priorities, skewed incentive and reward systems, a culture of denial, behavioural and social forces – cultural elements).

Isolation is more common in older age (82). Digital technology may foster contact with the outside world (83) and facilitate physical exercises led by a professional instructor. In an earlier study (84), focus group participants (mean age 83) were willing to adopt new technologies when their usefulness surpassed feelings of inadequacy, despite concerns to do with society’s overreliance on technology, loss of social contact, and the complexity of technological devices. Higher computer anxiety predicts lower use of technology (83). Our qualitative study explored the acceptability and usability of online physical exercises as a possible tool for improving the health and well-being of older adults (77). The findings supplement previous studies investigating how older adults view new technologies (77, 85). Our study goes one step further and researches not only how older adults view new digital technology but also how the online environment can hinder/promote the motivation of older adults to engage in physical exercises.

As for management skills, our findings show that the skills and abilities of managers/trainers leading a traditional workout are insufficient, meaning they must be improved for leading the virtual environment and virtual workout teams (13). Communication has to be clear, precise, transparent and comprehensive. The trainers reported that coping with musculoskeletal problems and adapting to the individual exercises was hard. It was necessary to repeat the same movement patterns over and again because the participants found it difficult to understand and forgot them between one training unit and the next. Controlling the execution of exercises was another of the more challenging challenges while leading the exercises. To inspire and motivate employees, a manager/trainer (coach) of a virtual workout team must create a positive atmosphere and show great understanding of individual older group members as they may find the workout conditions at home to be very different and difficult. Trainers tried to explain to participants why exercise benefits them, and the importance of socialising after the exercise to raise their motivation when they noticed that motivation was dropping. Interestingly, the trainers did not notice any difference in motivation between the group exercising in the gym and the online group. Both groups were highly motivated, as was also evident in the focus group. A good manager/coach of a virtual workout team must find a way to involve the participants. Otherwise, they will feel isolated, lose the sense that they belong to the workout team, or may not have enough information and knowledge and lose sight of the ‘big picture’ of the workout team’s physical exercise processes and common goals.

Managing a virtual workout team requires managers/coaches to undergo specialised training in managing remote workout teams. For the managers/coaches with years of experience leading conventional workout groups, this was quite a challenge especially since, besides preparing physical exercises for older adults, they also had to consider their level of technical skills as well as the older adults’ digital technology skills. They needed to maintain the motivation and satisfaction of older adults who they had not actually seen in person (13). The trainers think the online training did not take off because people still value personal relationships. Perhaps in the future, when the current younger generations are older, this will change. The key factors are the organisation and provision of information concerning the individual movements of older adults. Care must be taken to show the proper execution of the exercise, including at the right angle, and to describe it correctly so that the older trainee can perform it more easily and appropriately. Given that life expectancy is growing and that the older adults are also increasingly computer literate, the trainers see great potential in online exercising. This would also give access to a larger number of people who live in more remote areas and cannot drive to fitness centres where exercising usually takes place. At the same time, this is a good measure for the older adult’s exercising if the COVID-19 pandemic recurs or another similar long-term limit on association is imposed. In particular, it is necessary to ensure the training programme and the individual training units have a good structure, where the exercises are clearly and simply displayed and explained, and to provide the best possible feedback to the trainees about how they are performing the exercises.

Managers should be aware of members feeling less of a sense of belongingness when exercising with the support of online tools. The coaches of online physical exercises (compared to face-to-face physical exercises) should motivate and encourage virtual older members more often and show greater empathy and understanding of the different workout environments. A recent study by Vučković and Kajtna (86) found that older adults exercise because they want to recover, stay healthy, control their weight, and remain mobile (in contrast with young adults who crave social recognition and exposure but also wish to become stronger and compete with others while doing exercises). Participants who played competitive sports when they were young exercise in their older age because they like to exercise and compete. They also want to be socially recognised and praised more than non-competitors, who primarily exercise for health reasons. Single people have statistically significantly different motivations to exercise than people in a relationship, largely for affiliation and social recognition. In contrast, married people exercise mostly for health reasons. Employment status has an important impact on exercise motivation, as does education. People with higher education levels exercise more for health reasons, whereas less educated people exercise more for social recognition and for the challenge (86). In our sample, most respondents were high school graduates and enjoyed the good atmosphere of the face-to-face exercise the most. However, staying in shape was another very important factor for them to continue practising even after the 3-month pilot ended.

In addition, new roles may emerge in an online physical exercise setting compared to face-to-face physical exercising, such as a member responsible for providing information, a member responsible for planning exercises, a member responsible for inviting people to informal conversations etc. The learning curve in technology adoption is much steeper for older adults than younger ones, who naturally adopt new technologies faster (13). We thus encourage coaches to consider appointing a person to specifically engage with new technology (i.e., technology implementation advice and help) that may be crucial for older adults to overcome the technology barrier. The lack of social face-to-face contact holds important psychological consequences. Accordingly, the organisers of online physical exercises may consider engaging psychologists to help lower the psychological constraints of exercising in a virtual environment.

The well-integrated management of older adults’ exercise is essential for developing a system that supports their physical fitness. The WHO is physical exercise guidelines refer to at least 150–300 min of moderate-intensity aerobic physical activity or 75–150 min of high-intensity aerobic physical activity per week. At least twice a week, there should be strength exercises for the main muscle groups, and at least three times a week, a variety of balance exercises. Isometric or static exercises for developing strength, balance and mobility are the most recommended by trainers for older adults. In addition, aerobic exercises and exercises to strengthen the stabilisers of the trunk are welcomed, where 3–4 times a week for 1 h is the optimum exercise duration. Trainers’ advice for other trainers who wish to create physical exercises for older adults is to design a progressive training programme, choose simple exercises for the large muscle groups including movement patterns that mimic everyday movements, and use balance exercises to help prevent against falls. A great deal of patience and understanding is called for.

Workplace exercising as a preventive measure is excellent during the working day as a person can disconnect from work a little and socialise with their colleagues outside of their work circle, which has a good psychological impact. Physically you stretch, exercise, and raise your heart rate somewhat. One company we contacted for an interview to obtain feedback on the workplace exercises stated that they have 3–4 promoters who take turns every day to implement a 10-min exercise routine for the employees, which while it is intended for all of them, it is not mandatory. At the workplace, in October 2022 they started with an organised exercise routine, and a short break to exercise and stretch well during the 8-h workday (mainly while sitting). The focus is on the spine, shoulder girdle, neck, and other parts (79).

Older adults need support if they are to maintain ‘normal’ integration in a contemporary society that is technically advanced. Physical fitness enables them to be part of society and to use technological advances, such as micro-mobility. Older adults have shown rising micro-mobility-related mortality and rate of incidence in China, India and the United States (87). Promoting active ageing and healthy mobility among seniors is a global challenge. The recent diffusion of e-bikes, mobility scooters and electric tricycles points to new opportunities for older adults to move around with less effort and much later in life than they previously imagined. This was sometimes raised as a problem in our study. Older adults might not feel comfortable using a car anymore. However, they still wish to be active and use e-bicycles to travel to face-to-face exercises or other gatherings. As electric micro-mobility makes it easier for older people to stay active and reduce their isolation, they might, in turn, require cities and communities to implement better, more people-centric infrastructure to support them, delivering great benefits for residents and commuters of all ages (88). Education plays a vital role in changing the stereotypes, professionalism, and implementation of exercise tailored to older adults’ needs. It is a key mechanism for promoting exercise among older adults. Students who had completed the course “*Exercise of older adults persons with some chronic diseases, persons with acute and/or chronic injuries of locomotor apparatus 1*″ at the Faculty of Sport at the University of Ljubljana who carried out the training for older adults in the SI4CARE pilot know the characteristics of planning, organising and conducting exercises for persons with special needs: exercise selection, methods of loading, periodisation, control of the exercises’ effects. They are able to programme and conduct exercise programmes for special groups of individuals [older persons, disabled, chronic diseases, injuries of the muscular-skeletal system; (89)]. Exercise for the conditioning of special groups is a complex and specific management process involving exercise planning, organisation, leading and controlling. The development of important basic motor abilities is based on exercises for strength, power, balance, endurance, coordination, flexibility, kinaesthesia, eccentric exercises, and plyometric exercises in rehabilitation. Educating the trainers of older adults entails examples of exercises complexes for injuries (spine, knee, ankle, shoulder etc.) and the analysis of exercise effects. Exercise for older adults is composed of a management process with a detailed planning process. Exercises can be performed in different environments (89): (a) indoor (group exercises face-to-face, online exercises, adapted sports); and (b) outdoor (walking, running, polygons, exercise tracks, different organisation modes, swimming etc.).

Trainers will need to take on new responsibilities, roles and functions, while future research could help identify which of these are required to accelerate the digital transformation of physical exercises for older adults (90). Such research could focus on a particular type of communication channel and apps offering online physical exercises for older adults. Perhaps the most demanding issue while discussing online exercising still open for future research is the transfer of haptic feedback between trainer and trainee, which is an essential part of individual training in-person that allows the trainer to manually correct the execution of an exercise or check the tension of groups of muscles that should be activated during a given exercise.

## Data availability statement

The original contributions presented in the study are included in the article/supplementary material, further queries can be directed to the corresponding author(s).

## Ethics statement

The studies involving humans were approved by the Ethical Committee for Scientific Research from the School of economics and business University of Ljubljana confirms that the application for conducting the research entitled “Older Adults’ Perceptions of the (Online) Physical Exercises Management,” whose responsible researchers are Judita Peterlin (SEB UL), Vlado Dimovski (SEB UL), Simon Colnar (SEB UL), Anamarija Kejžar, (SEB UL), Bojan Blažica (Institute Jožef Stefan) is adequate and complete and gives his consent to the implementation of the proposed research. The studies were conducted in accordance with the local legislation and institutional requirements. The participants provided their written informed consent to participate in this study. Written informed consent was obtained from the individual(s) for the publication of any potentially identifiable images or data included in this article.

## Author contributions

JP: Conceptualization, Formal analysis, Funding acquisition, Investigation, Methodology, Project administration, Supervision, Validation, Writing – original draft, Writing – review & editing. VD: Conceptualization, Formal analysis, Funding acquisition, Project administration, Supervision, Validation, Writing – review & editing. SC: Conceptualization, Data curation, Project administration, Resources, Validation, Writing – original draft, Writing – review & editing. BB: Conceptualization, Investigation, Methodology, Resources, Software, Supervision, Writing – original draft. AK: Visualization, Supervision, Writing - original draft, Writing – review & editing.
